# Lessons from Injury: How Nerve Injury Studies Reveal Basic Biological Mechanisms and Therapeutic Opportunities for Peripheral Nerve Diseases

**DOI:** 10.1007/s13311-021-01125-3

**Published:** 2021-09-30

**Authors:** Peter Arthur-Farraj, Michael P. Coleman

**Affiliations:** Department of Clinical Neurosciences, John Van Geest Centre for Brain Repair, University of Cambridge, Robinson Way, Cambridge, CB2 0PY UK

**Keywords:** Wallerian degeneration, Programmed axon death, Repair Schwann cell, NMNAT2, SARM1, C-JUN, Regeneration, Neuregulin

## Abstract

Since Waller and Cajal in the nineteenth and early twentieth centuries, laboratory traumatic peripheral nerve injury studies have provided great insight into cellular and molecular mechanisms governing axon degeneration and the responses of Schwann cells, the major glial cell type of peripheral nerves. It is now evident that pathways underlying injury-induced axon degeneration and the Schwann cell injury-specific state, the repair Schwann cell, are relevant to many inherited and acquired disorders of peripheral nerves. This review provides a timely update on the molecular understanding of axon degeneration and formation of the repair Schwann cell. We discuss how nicotinamide mononucleotide adenylyltransferase 2 (NMNAT2) and sterile alpha TIR motif containing protein 1 (SARM1) are required for axon survival and degeneration, respectively, how transcription factor c-JUN is essential for the Schwann cell response to nerve injury and what each tells us about disease mechanisms and potential therapies. Human genetic association with NMNAT2 and SARM1 strongly suggests aberrant activation of programmed axon death in polyneuropathies and motor neuron disorders, respectively, and animal studies suggest wider involvement including in chemotherapy-induced and diabetic neuropathies. In repair Schwann cells, cJUN is aberrantly expressed in a wide variety of human acquired and inherited neuropathies. Animal models suggest it limits axon loss in both genetic and traumatic neuropathies, whereas in contrast, Schwann cell secreted Neuregulin-1 type 1 drives onion bulb pathology in CMT1A. Finally, we discuss opportunities for drug-based and gene therapies to prevent axon loss or manipulate the repair Schwann cell state to treat acquired and inherited neuropathies and neuronopathies.

**Supplementary Information:**

The online version contains supplementary material available at 10.1007/s13311-021-01125-3.

## Introduction

Traumatic injury has long been used to study peripheral nerve degeneration and regeneration [1, 2], in part because morphological similarities with peripheral neuropathies and other nerve disorders suggested similar mechanisms and the potential to inform therapies [3]. More recently, the advent of molecular biology and the use of spontaneous and induced mutants in mice and other species have revealed similar molecular mechanisms underlying injury and disease. The central theme of this review is that both the axon-intrinsic degeneration mechanism after nerve injury and the Schwann cell response leading to nerve repair have been revealed mostly by nerve injury and genetic modification studies but are proving highly relevant also in peripheral nerve disorders, including many that do not involve traumatic injury. Good drug targets and genetic therapies have emerged in both fields that promise to underlie future advances in the treatment of peripheral neuropathies.

**Fig. 1:**
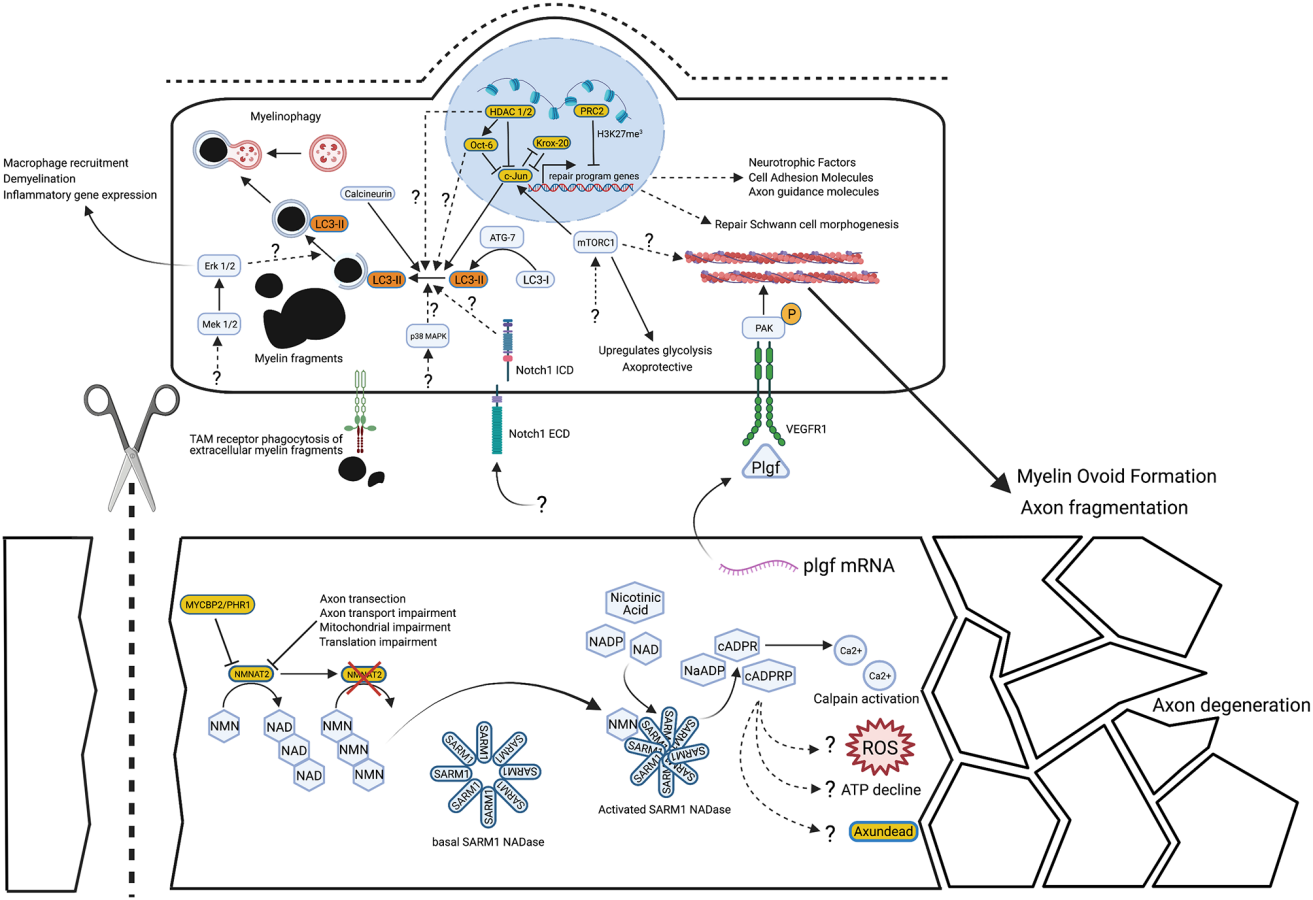
Overview of the molecular mechanisms within the axon and the Schwann cell during Wallerian degeneration. Upon nerve transection, the axonal transport of NMNAT2 is interrupted, and NMNAT2 already present in axons is degraded in a PHR1- and proteasome-dependent manner. Conversion of NMN to NAD by NMNAT2 is halted so NMN builds up inside the axon. NMN binds the SARM1 octamer, causing a conformational change and its activation. SARM1 activity generates cyclic ADP-ribose (cADPR) from NAD but also other products from nicotinamide adenine dinucleotide phosphate (NADP) and other substrates, such as nicotinic acid adenine dinucleotide phosphate (NaADP) and 2′-phospho-cyclic ADP-ribose (cADPRP). It is incompletely understood how SARM1 activation leads to further downstream steps in the axonal degeneration pathway, such as calcium release, ROS generation, ATP decline and the role of the molecule Axundead. The timings of the activation of the molecular pathways involved in the Schwann cell injury response in relation to those that regulate the axon degeneration machinery have not been fully delineated. It is likely that the majority of the Schwann cell injury response occurs during or slightly after axon degeneration has been executed. During axon degeneration, placental growth factor (Plgf) is released from axons and activates VEGF receptors leading to constriction of actin filaments in the Schwann cell, which helps break up axon fragments. It is possible that mTORC1 activation contributes to this process. Within the nucleus, c-JUN upregulation mediates a substantial amount of the Schwann cell response to nerve injury, especially repair program gene expression, cell shape change forming repair Schwann cells, upregulation of myelinophagy to aid in myelin sheath removal and repression of the myelin program through inhibition of Krox-20 function. Other pathways that aid myelin clearance include calcineurin, MEK-ERK, Notch and P38 MAPKinase pathway activation, though their full mechanism is not completely understood. Furthermore, TAM receptor phagocytosis also contributes to myelin clearance. Within the nucleus, both OCT6 and HDAC1/2 repress c-JUN function and the polycomb repressive complex 2 (PRC2) represses a number of other repair program genes. Broken lines with question marks highlight a hypothetical association or an unknown quality. Created with BioRender.com

A transected nerve degenerates by Wallerian degeneration [2]. For the first 150 years after Wallerian degeneration was first described, we knew very little of its molecular mechanism, but in the last 20 years, this has changed dramatically. Lubinska in the 1970s and early 1980s had moved beyond Waller’s original concept that injured axons die passively because they lack “nourishment” by the soma, suggesting instead a specific, soma-derived inhibitor of axon degeneration and of the Schwann cell response. She proposed that this inhibitor is delivered by anterograde axonal transport and becomes depleted distal to a site of injury [4, 5]. Today, the best known match for this inhibitor is NAD-synthesizing enzyme nicotinamide mononucleotide adenylyltransferase 2 (NMNAT2) [6], because it is essential for axon growth and survival [7], quickly depleted distal to an axon injury [6] and functionally similar to a much more stable, aberrant fusion protein that strongly delays Wallerian degeneration [8]. Further understanding of the degenerative mechanism that NMNAT2 blocks has been largely driven by *Drosophila* genetics followed by confirmation in mammals [9–11]. It seems Wallerian degeneration is a remarkably well-conserved process involving enzymes that are functionally interchangeable between mammals and flies. The central execution step involves Toll-like receptor (TLR) adapter protein sterile alpha and TIR motif containing protein 1 (SARM1) [10], which has unexpected, intrinsic NADase and other enzyme activities required for its prodegenerative role [12]. The existence of a protein whose activated form kills axons but which is inhibited in healthy axons through the actions of other proteins has led to the concept of a programmed axon death mechanism (Fig. 1) that underlies both Wallerian degeneration after nerve injury and axon loss in a wide range of inherited, toxic and metabolic disorders.

Ramon y Cajal demonstrated the regenerative ability of peripheral nerves and postulated that it was likely some property of the distal stump that attracted axons to regenerate through it. Cajal further described in great detail the fragmentation of myelin sheaths, Schwann cell proliferation and lateral infiltration of Schwann cell tubes with haematogenous macrophages [1]. Progress was made in the 1960s with the application of electron microscopy, which allowed detailed visualisation of the cellular events that occur after nerve injury. However, up until the last two decades, there has been much debate over three questions. Firstly, do Schwann cells actively respond to nerve injury through a controlled molecular mechanism or do they passively revert to an immature phenotype when they lose axonal-derived differentiation signals following axon degeneration? Secondly, do Schwann cells contribute to myelin clearance or is all myelin and axonal debris removed by macrophages and other immune cells? Thirdly, how important are Schwann cells for axon regeneration and functional nerve repair? Through use of mouse conditional knockout technology, we now know that Schwann cells actively respond to nerve injury and this process is regulated by a number of genes, but expression of the transcription factor c-JUN is crucial [13, 14] (Fig. 1). We also know that Schwann cells do not revert to an immature phenotype after injury but are instead reprogrammed to an injury specialised cellular state, termed, repair Schwann cells [13, 15]. Repair Schwann cells use a form of macroautophagy, termed myelinophagy to clear myelin debris after injury, alongside myelin clearance by haematogenous macrophages [16, 17]. Finally, if repair Schwann cell formation is disrupted, then sensory and motor axon regeneration is significantly slowed, a substantial proportion of neurons die and the PNS repair process is permanently arrested [13].

In the last decade, there have been substantial advances in the understanding of the cellular and molecular mechanisms that govern axon degeneration and the Schwann cell injury response. Furthermore, there has been recent progress in linking some of these underlying mechanisms to neurological disease and to developing therapies to both protect from axon loss and to promote axons to regenerate once they have been damaged. In this review, we will highlight the current understanding of the signals that govern the axon intrinsic mechanism of degeneration and the Schwann cell response to PNS injury and repair. This includes what is known from studies using rodents, fish and flies about the molecular pathways governing axon degeneration, those regulating repair Schwann cells, demyelination and the axon extrinsic mechanisms of regeneration. We will not discuss the axon intrinsic mechanisms of regeneration, which are reviewed elsewhere [18–20]. It is important to remember that many additional cell types play significant roles in PNS injury and repair too, including cells of the innate and adaptive immunity, satellite glia in the dorsal root ganglion (DRG), perineurial glia, endoneurial fibroblasts/tactocytes and endothelial cells. These topics are beyond the scope of this review and are discussed in detail elsewhere [21–26]. We will then comment upon recent research linking genes involved in regulating axon degeneration and repair Schwann cells to PNS diseases. Finally, we will discuss the current translation of these fundamental biological mechanisms into therapies to both protect against axon loss and promote axon regrowth in the diseased or injured PNS, and some immediate, outstanding questions for these fields to answer.

## Basic Biology of Injury-Induced Axon Degeneration

Today’s detailed knowledge of the molecular mechanism of programmed axon death stems from the discovery in 1989 of an overtly normal strain of mice in which a spontaneous mutation delayed the axon degeneration distal to a nerve injury by tenfold [27]. Instead of the normal latent phase of around 36 h, during which substantial Schwann cell morphological responses discussed below begin, the distal stump remains intact for 2–3 weeks [28]. Both PNS and CNS axons of these Wallerian degeneration slow (*WLD*^S^ mice are protected; they remain functionally competent for much of their extended survival time if an action potential is artificially evoked in the distal stump [8, 27], and the molecular mechanism is clearly distinct from programmed cell death by apoptosis [29, 30]. However, the principle of a self-destructive mechanism blocked by upstream regulators and activated by multiple, diverse stimuli does indeed mirror that of apoptosis. Why it should be evolutionarily beneficial to have a self-destruct mechanism for rapid axon loss remains unclear but possible explanations including preventing spread of pathogens around the nervous system by axonal transport [31] and promoting subsequent nerve repair. This seems consistent with the slower degeneration in the mammalian CNS, although there are indications that this mostly reflects slower removal of myelin debris [32], while axon degeneration itself is only marginally slower [33].

The discovery that the *WLD*^S^ mutant gene encodes an NAD synthesising enzyme [8] of the NMNAT family that is partially targeted into axons [34] began a series of findings, still going on today, of how closely axon survival is linked to NAD-related metabolism (Fig. 1). Overexpression of other NMNAT isoforms was found to protect injured axons too, at least if these are axonally targeted and sufficiently stable [35–38]. However, when endogenous NMNATs are removed, the only one whose loss causes an axonal phenotype is NMNAT2 [7, 39, 40] ([41] p. 3).

The remarkable finding that murine *WLD*^S^ protects injured axons when ectopically expressed in *Drosophila* validated the use of *Drosophila* as an experimental organism for further genetic analysis [42, 43]. This led to the identification of dSarm, the *Drosophila* orthologue of SARM1, as a protein required for injured axons to undergo rapid axon degeneration, and confirmation that mammalian SARM1 is also an essential effector of Wallerian degeneration [10]. An RNAi-based screen in mouse neurons subsequently confirmed this finding [44], and further research revealed an unexpected NAD degrading enzyme activity of SARM1 [12, 45]. SARM1 is also an nicotinamide adenine dinucleotide phosphate glycohydrolase (NADPase) and has base exchange activities that are sometimes even dominant over its NADase activity [46, 47]. Any one of these activities could drive or contribute to axon degeneration, although most attention has so far focussed on NADase. NMNAT2 was found to be a upstream negative regulator of SARM1, in fact *Sarm1* deletion in mice completely rescues the otherwise perinatal lethal *Nmnat2* null phenotype in which long axons fail to grow [48, 49]. This, and a similar, recent finding of complete protection by *Sarm1* deletion from a neurotoxin [50, 51], shows the full protective capacity that could be achieved by effective targeting of SARM1, at least in disorders where programmed axon death is activated very specifically.

Considerable recent progress has begun to reveal how NMNAT2 holds the SARM1 enzyme activity at basal levels. Loss of NMNAT2 from injured axons leads to accumulation of its substrate, NMN, which was found to promote axon degeneration [52]. Sequestering NMN using an ectopically expressed bacterial enzyme, NMN deamidase, is highly protective [53]. For several years, this led to competing hypotheses regarding whether axons die from NAD depletion or from accumulation of its precursor NMN to toxic levels [54] until these were unified by the exciting discovery that NMN is an activator of SARM1 NADase [47] (Fig. 1). Thus, accumulation of NMN after NMNAT2 loss does not just accompany NAD depletion due to loss of its synthetic enzyme; it actually drives NAD depletion even faster by increasing NAD degradation. In the latest developments, NAD has been found to oppose the activating effect of NMN through binding of the same allosteric site in the inhibitory ARM domain, countering one another at physiological levels of each [46, 55]. Another, more potent activator has also been identified [50, 51]. Vacor mononucleotide (VMN), an analogue of NMN and a metabolic product of the disused neurotoxin vacor, was found to bind and activate SARM1 with around twice the potency of NMN, killing neurons and their axons, suggesting this is the likely basis of vacor toxicity [56]. With all three structures now available [50, 51, 55, 57], these findings greatly facilitate rational drug design targeting SARM1 regulation.

Some additional progress has been made upstream of NMNAT2 and downstream of SARM1. NMNAT2 is targeted to axonal transport vesicles by palmitoylation, which unexpectedly lowers its stability and its capacity to protect injured axons [35, 36]. A partial explanation is that NMNAT2 also exists in a separate, soluble pool [35, 36, 58] and that turnover of the vesicular and soluble proteins is regulated by different proteins. The MYCBP2(PHR1)/FBXO45/SKP1A ubiquitin ligase complex regulates turnover of the vesicular form, and kinases DLK and LZK regulate the half-life of the soluble form. Interestingly, inhibition of these proteins, or corresponding gene deletion, is also protective [9, 59]. Stathmin-2 (STMN2) has an as-yet undefined role as another, albeit weaker inhibitor of programmed axon death [60] which may be important in the context of ALS (see below). Its many similarities to NMNAT2, including being targeted by palmitoylation to the same vesicle population and being turned over by the same enzymes [58], suggest this is the most likely point at which it impacts the pathway, although this remains to be determined.

Downstream of SARM1, the loss of NAD, and subsequently of ATP, is not the only important consequence. The loss of NADP, and consequently of NADPH, is likely to limit the capacity for reactive oxygen species (ROS) buffering, especially as SARM1 NADPase is also activated by NMN [46], and there is SARM1-dependent accumulation of calcium [61] that may drive degeneration through calpains [62], likely due to the several calcium mobilising products of NAD cyclisation and base exchange (Fig. 1). The *Drosophila* protein Axundead also has a poorly understood but essential role downstream of dSarm [63] that may yet fit with any of these mechanisms (Fig. 1).

## The Schwann Cell Response to Nerve Injury

Myelinating and non-myelinating Schwann cells have major roles in both the degeneration and regeneration phase of nerve injury. Schwann cells react early to nerve injury with changes in gene expression; however, it is still not clear whether these changes occur before or at the time of axon degeneration [14, 64]. Several studies, conducted first in zebrafish and later in mice, have shown that Schwann cells participate in the breakup of the axon during the process of axon degeneration [65–70]. This process involves the formation of constricting actomyosin spheres and partially requires placental growth factor signalling from the axon, the vascular endothelial growth factor receptor (VEGFR) on Schwann cells, activation of mechanistic target of rapamycin (mTOR) and potentially calcineurin B in Schwann cells [65, 69, 71, 72]. Furthermore, it has recently been shown that Schwann cells upregulate glycolysis after injury and that this process may actually help to protect axons for a short period after injury prior to axon degeneration [65].

During or slightly after the process of axon degeneration, though the exact timing is still unknown, Schwann cells undergo a remarkable biochemical and morphological transformation into repair Schwann cells [13–15]. This conversion can be described as a reversible injury-induced change of cellular state, termed adaptive cellular reprogramming. This is similar to other adult mammalian cellular responses to injury, such as fibroblast to myofibroblast conversion in wound healing, as well as the PNS neuronal upregulation of an axon regeneration program [73]. As part of this transition, repair Schwann cells ingest a proportion of their own myelin sheaths using a form of macroautophagy, termed myelinophagy [17]. Lipidated LC3 (LC3-II), a marker of autophagosomes, is strongly expressed in demyelinating Schwann cells, in vitro and in vivo, in addition to many autophagy machinery genes, such as *Atg7*. When *Atg7* is specifically inactivated in Schwann cells, autophagy and thus myelin clearance after nerve injury is significantly perturbed [17, 74, 75]. Additionally, Schwann cells also use phagocytosis through TAM receptors and the necroptosis pathway to clear myelin debris [74, 76] (Fig. 1). The formation of repair Schwann cells also involves substantial morphological changes to myelinating and non-myelinating Schwann cells transforming into vastly longer, bipolar, branched repair Schwann cells that partially overlap with neighbouring cells within their basal lamina tubes, forming the bands of Büngner [15]. Correct formation of the bands of Büngner likely underlies efficient axon regeneration [13]. This potentially explains why PNS regeneration is more efficient after nerve crush compared to a full nerve transection as a crush injury maintains continuity of the Schwann cell basal lamina tubes between proximal and distal sites [77]. On the contrary, when a nerve is fully transected a multicellular bridge is formed from Schwann cells, fibroblasts, perineurial cells, blood vessels, macrophages and regenerating axons [22]. A number of factors have specific roles in modulating the Schwann cell phenotype specifically in the nerve bridge,these include SOX2, TGFβ1, Robo signalling and ephrin-B/EphB2 signalling. These are reviewed elsewhere [22, 78].

Repair Schwann cell formation involves large-scale changes in gene expression. Genes involved in myelin differentiation are suppressed, and instead there is upregulation of genes involved in a repair program [79]. The repair program broadly comprises of (1) re-expression of some developmentally expressed genes such as *N-cadherin*, *Sox-2*, *c-Jun*, *p75ngfr* and *Gfap*, which are normally repressed in myelinating Schwann cells; (2) expression of cytokines and chemokines, such as tumour necrosis factor-α (TNF-α), interleukin-6 (IL-6), IL-1α/β, leukaemia inhibitory factor (LIF) and monocyte chemoattractant protein-1/CCL2 (MCP-1); and (3) upregulation of genes and proteins that are important in promoting axon guidance and neuron survival, such as GDNF, BDNF, artemin, NT3, sonic hedgehog (SHH), semaphorins (*e.g. Sema4F*) and ephrins (e.g. *Epha5*) in addition to cell adhesion and matrix molecules such as integrins (e.g. *Itgb2*), collagens (*e.g. Col18a1*) and matrix metalloproteins (e.g. *Mmp17*) [13, 14, 64, 80].

The transition of a myelinating Schwann cell into a repair Schwann cell shares many similarities with the process of epithelial-mesenchymal transition (EMT) [14]. Myelinating Schwann cells represent an epithelial-like cell, since they have tight junctions, a basement membrane, cell polarity with an adaxonal and abaxonal membrane and express epithelial proteins such as E-cadherin, claudin-19, occludin and the polarity protein, PAR3 [81–85]. Their injury induced conversion into repair cells involves the formation of a more motile, proliferative and invasive cellular state, similar to cells of mesenchymal origin and express a number of EMT-enriched genes such as *vimentin*, *snail*, *Tgf-β1*, *Wt1*, *Met*, *Hmga2*, mir221 and mir222 [64, 86].

### Molecular Signals that Regulate Repair Schwann Cells, Remyelination and the Non-cell Autonomous Regulation of Axon Regeneration

**Fig. 2:**
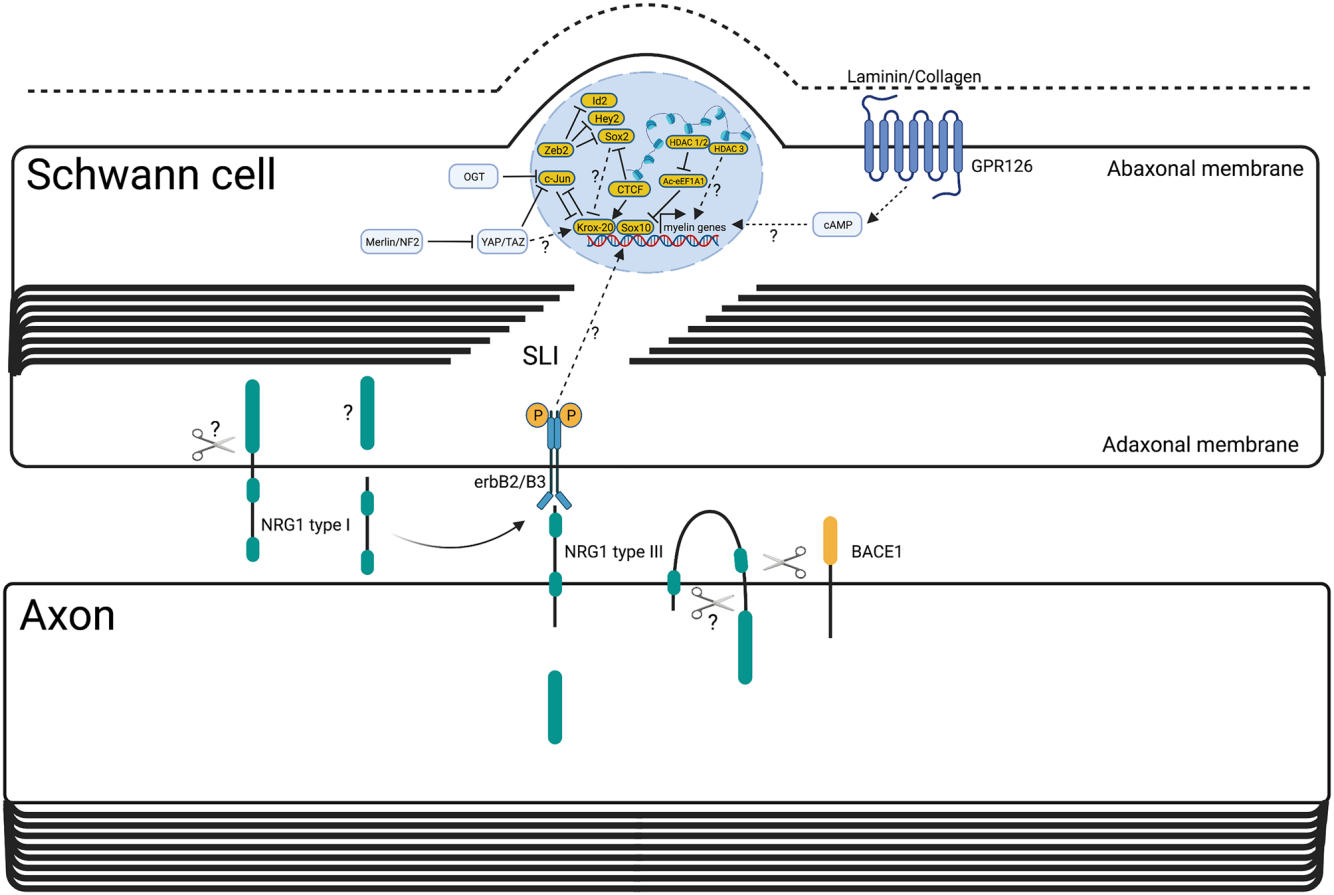
Overview of the molecular mechanisms of Schwann cell remyelination. Schwann cell remyelination is promoted by axonal signals centred around NRG1 type III and basal lamina signalling via the g-protein coupled receptor, GPR126, similar to myelination during development. One distinct molecular difference from development is that Schwann cell derived soluble NRG1 type I also contributes to remyelination. Certainly macroscopically, remyelination leads to thinner myelin sheaths and shorter internodal distances compared to developmental myelination. C-JUN is an inhibitor of remyelination and OGT, through direct O-GlcNAcylation, represses c-JUN function to allow remyelination to proceed. YAP/TAZ is required for remyelination, in addition to other important regulators of myelination, such as CTCF, ZEB2, HDAC1/2 and HDAC3. SLI = Schmidt-Lanterman incisure. Broken lines with question marks highlight a hypothetical association or an unknown quality. Created with BioRender.com

We will briefly summarise the major transcription factors, signalling pathways and epigenetic factors that regulate repair Schwann cells, but this topic has been comprehensively reviewed elsewhere [14] (Figs. 1 and 2). The transcription factor, c-JUN, was identified as a central regulator of repair Schwann cells, controlling demyelination/myelinophagy, expression of many repair program genes, such as *Shh*, *Bdnf* and *Gdnf*, axon regeneration, motor and sensory neuron survival and functional recovery [13, 17, 87] (Fig. 1). C-JUN expression is suppressed in Schwann cells in the adult nerve and is strongly upregulated after nerve injury [88]. C-JUN acts as an inhibitor of myelination and re-myelination, and its timely O-GlcNAcylation is necessary to inhibit its activity and promote remyelination of regenerated axons [88–90]. Additionally, the POU domain transcription factor, OCT-6 is upregulated by Schwann cells after injury and appears to repress c-JUN induction and delay demyelination and axon regeneration [91] (Fig. 1). Another important transcription factor is STAT3 which promotes the long-term survival of Schwann cells after nerve injury, in addition to maintaining the expression of c-JUN and other repair program genes [92].

The transcription factor ZEB2 and two transcriptional activators of the Hippo signalling pathway, YAP and TAZ, are required for Schwann cell remyelination after injury but not for the initial formation of repair Schwann cells, myelin clearance or c-JUN upregulation (Fig. 2). ZEB2-deficient Schwann cells fail to remyelinate, and it is postulated that this is due to maintained expression of myelination inhibitors, such as *Sox2*, *Hey2* and *Id2*. While it is uncertain whether physiological levels of YAP/TAZ directly regulate c-JUN in Schwann cells, in vivo, YAP/TAZ does appear to be required for eventual c-JUN downregulation during remyelination [93–96]. Furthermore, the tumour suppressor protein, merlin, is important for timely c-JUN upregulation though not for demyelination and is crucial for adequate axonal regeneration and proper remyelination after injury. In the absence of merlin, YAP is aberrantly overexpressed after nerve injury, and deletion of *Yap* is sufficient to rescue the regenerative deficit in merlin null mice [97].

Epigenetic factors such as chromatin remodelling enzymes, non-coding RNAs and DNA methylation also regulate the Schwann cell injury phenotype [14, 98]. Nerve injury induces demethylation of the repressive histone mark, H3K27 trimethylation, at enhancers of a number of repair program genes (Fig. 1). Importantly activity of the histone demethylases, KDM6B/KDM6A, appears not to be involved [99, 100]. Concurrently, repair program gene promoters gain the active H3K4 methylation mark, which is also postulated to drive their expression [101]. The polycomb repressive complex 2 (PRC2) acts as a histone methyltransferase and is responsible for repressing expression of a number of Schwann cell injury genes such as *Fgf5*, *Shh*, *Sema4f*, *EphA5*, *Olig1*, *Runx2*, *Nrg1* and *Mmp17*, likely through H3K27 trimethylation [102] (Fig. 1). Deletion of the Eed subunit of PRC2 in Schwann cells leads to aberrant upregulation of a subset of repair program genes in the uninjured state and early after injury. However, Eed appears to be largely dispensable for repair Schwann cell formation, since demyelination, c-JUN expression and remyelination are normal, and there is only a temporary delay in axonal regeneration, in its absence [102]. These findings suggest that H3K27 demethylation of enhancers may work in parallel with upregulation of c-JUN-dependent genes in Schwann cells after nerve injury.

The chromatin remodelling enzymes, histone deacetylases (HDACs) have been shown to have several roles in Schwann cells after injury. Deletion of *Hdac1/2* in Schwann cells leads to accelerated myelin clearance and axon regeneration but impaired remyelination, whereas *Hdac3* deletion has no effect on demyelination or regeneration but leads to exaggerated remyelination [91, 103]. *Hdac 1/2* deletion impairs OCT-6 upregulation after injury and causes exaggerated levels of c-JUN expression, which may explain why there is faster demyelination and axon regeneration [91] (Fig. 1). HDAC2 has been shown to de-acetylate eukaryotic translation elongation factor 1 alpha 1 (eEF1A1), inactivating its ability to shuttle SOX10 out of the nucleus, thus allowing remyelination to proceed [104] (Fig. 1). HDAC4 is known to supress expression of c-JUN in Schwann cells in development, but it’s function has not yet been studied after nerve injury [105]. Additionally, the CCCTC-binding factor (CTCF) has recently been shown to be crucial for both PNS myelination and remyelination after injury, likely through modifying the chromatin accessibility of myelin gene promoters and enhancers, in particular, *Krox-20 *[230]. MicroRNAs appear to help repress the expression of some repair program genes during development and also promote remyelination [106, 107], whereas the role of long non-coding RNAs remains largely unknown [14, 231]. Finally, there are no global changes in the DNA methylome after nerve injury arguing against a major role for CpG methylation in regulating the repair cell phenotype. It is unknown whether DNA hydroxymethylation or hemimethylation play roles in Schwann cells however [64, 108].

A number of signalling pathways are activated in Schwann cells soon after injury [14]. The mTOR pathway is activated rapidly after nerve trauma, and, conditional removal of mTORC1 in Schwann cells leads to a delay in demyelination, slower c-JUN upregulation and impaired upregulation of glycolysis [65, 109] (Fig. 1). The Notch pathway has a major role in controlling the rate of demyelination in injured nerves though it remains unknown whether it regulates repair Schwann cell formation, c-JUN expression and axon regeneration [110] (Fig. 1). The Raf-MEK-ERK mitogen-activated protein kinase pathway (MAPK) is important in regulating cytokine and chemokine expression by Schwann cells, macrophage influx into the nerve, demyelination after injury and may also play a role in regulating blood-nerve barrier integrity, in addition to axon regeneration rates [111–114] (Fig. 1). It remains uncertain whether the Raf-MEK-ERK pathway works in parallel to or mainly upstream of c-JUN controlled events within repair Schwann cells. Furthermore, ERK1/2 also has differing roles in myelination and remyelination, which is reviewed in more detail elsewhere [14]. The p38MAPK and c-Jun-N-terminal kinase (JNK) pathways are also both activated after nerve injury [88, 115] (Fig. 1). Inactivation of p38α, the major p38MAPK isoform expressed in the PNS, demonstrated slower myelin clearance after injury but no effect on macrophage influx or axonal regeneration, whereas the role of the JNK pathway in Schwann cells in vivo has not been studied yet [116]. Additionally, Schwann cell RalGTPases have been shown to regulate remyelination and motor neuron reinnervation and calcineurin B has recently been shown to regulate myelinophagy, independently of c-JUN expression [72, 117].

Neuregulin-1 (NRG-1) has 15 different isoforms and multifaceted roles in PNS development and repair [118]. Membrane bound NRG-1 type III is expressed on axons, though Schwann cells upregulate expression of NRG-1 type I after injury [119, 120]. Axonal NRG-1 and the erbB receptor complex expressed on Schwann cells appear largely dispensable for demyelination and repair Schwann cell formation [121–123]. Axonal NRG-1 and Schwann cell erbB signalling does play a role in influencing the rate of axonal regeneration, and both axonal NRG-1 type III and Schwann cell produced NRG-1 type I regulate remyelination after nerve injury [120, 122–124] (Fig. 2). Additionally, the transmembrane protease, BACE1, which cleaves NRG1, and the ERBB2 binding protein, Erbin, are both required for proper Schwann cell remyelination [125, 126]. The g-protein coupled receptor GPR126, expressed in Schwann cells, is important for both myelination and remyelination (Fig. 2). It also regulates, non-cell autonomously, macrophage influx into the injured nerve, axonal regeneration and terminal Schwann cell morphology as well as reinnervation of the neuromuscular junction, though its function in nerve injury does not require its prion protein ligand [127–129, 232]. GPR126 is dispensable for c-JUN upregulation and repair Schwann cell formation, however [128]. Schwann cells also upregulate many extracellular matrix (ECM) proteins and neurotrophic factors after nerve injury [130, 131]. Laminins, collagens, fibronectin and tenascins are all expressed by repair Schwann cells [131, 132]. Deletion of laminin γ1, *Col18a1* and tenascin C in the PNS all lead to defective axon regeneration [133–135]. Interestingly, post-translational modification of collagen by lysyl-hydroxylase 3 in Schwann cells in zebrafish underlies target selective regeneration [136].

Repair Schwann cells express a number of neurotrophins after injury, these include NGF which binds TrkA and p75NGFR receptors; BDNF and NT-4/5 which bind TrkB receptors; GDNF and artemin, which bind GDNF receptors; and the neuropoietic cytokines, CNTF and LIF, which bind their cognate receptors and signal through gp130 [130, 137]. Creation of a pan-neurotrophin mouse, which expressed a chimeric neurotrophin under the endogenous BDNF promoter, which is only active in repair Schwann cells after injury, accelerated motor and sensory axon regeneration [13, 138]. Use of NGF blocking antibodies demonstrated no reduction in nociceptor regeneration rates but did identify a role for NGF in injury-induced collateral sprouting [139–141]. Blocking BDNF function after injury led to reduced axon regeneration rates and a substantial reduction in myelinated axons numbers, however, BDNF haploinsufficiency is sufficient for normal sensory axon regeneration rates [142, 143]. Importantly, regeneration rates of Thy1.1 YFP labelled, sensory axons in NT-4/5 heterozygous and homozygous nerve grafts were substantially reduced [142]. GDNF and artemin are required for adequate motor neuron survival and regeneration, in addition to promoting the survival and function of a subset of large and small fibre DRG neurons [13, 87, 144–146]. Regarding the neuropoietic cytokines, CNTF acts as a lesion factor, released from myelinating Schwann cells, promoting the survival of motor neurons after injury, and LIF promotes the regeneration of sensory axons [147]. Finally, there are two additional factors produced by Schwann cells that have been shown to act in a similar way to the neurotrophins, the first is pleiotrophin and the other is sonic hedgehog, which promote PNS motor and sensory axon regeneration, respectively [13, 106, 148, 149].

## Relevance of Molecular Mechanisms Regulating Axon Degeneration and Repair Schwann Cell for PNS Diseases

A common approach to developing therapies for inherited and acquired peripheral nerve disorders is to identify the genetic and environmental causes and then find ways to block them. Examples of success include over 70 known genes for Charcot-Marie-Tooth disease [150] and the neurotoxicity of cancer chemotherapeutics such as vincristine, paclitaxel, bortezomib and oxaliplatin in chemotherapy-induced peripheral neuropathy [151, 152]. However, fully understanding and preventing the mechanisms these genes and toxins activate is more challenging. The well-characterised programmed axon death pathway, which can already be blocked, at least in animal models, offers an alternative approach of identifying specific human diseases and patients in which this contributes to axon loss. Animal model studies have strongly validated this approach [153], and human genetics now provides important opportunities for translation by identifying and functionally characterising naturally occurring human mutations in programmed axon death genes and testing for association with disease.

### Programmed Axon Death and Disease

The concept that degeneration after nerve injury may inform us about how axons degenerate in disease dates right back to Waller, who wrote of his observations on transected nerves: “it is particularly with reference to nervous diseases that it will be most desirable to extend these researches” [2]. When the discovery of delayed Wallerian degeneration in WldS mice raised the prospect of molecular understanding of the process in 1989, part of the impetus to identify the underlying genetic cause was a similar thinking, that this could have therapeutic implications for non-injury disorders. Indeed, the concept that toxins could produce an effective “chemical transection” of axons had recently been proposed by Bouldin and Cavanagh [154], and if that was their mode of action, then the degenerative mechanisms may be similar.

Confirmation that axons can die through the same WldS-sensitive pathway without physical injury first came in experiments with the cancer chemotherapeutics vincristine and paclitaxel [155, 156] in cell culture and mouse models of chemotherapy-induced peripheral neuropathies. Protection of axons exposed to these toxins by the WldS mutation clearly showed that physical injury was not necessary to activate this degenerative mechanism, strongly supporting the clinical relevance of understanding and blocking it. More extensive mechanistic similarities were later indicated by observations that *Sarm1* deletion also protects axons and alleviates the pain responses in these models [157, 158] and that axons expressing lower than normal NMNAT2 levels show enhanced vulnerability to vincristine [159]. The findings that WldS protects axons and alleviates symptoms in models of toxic disorders were quickly followed by reports of similar protection in mouse of genetic disorders, such as Charcot-Marie-Tooth 1B (CMT1B) involving myelin protein zero [160] and progressive encephalopathy with distal spinal muscular atrophy (SMA) involving biallelic mutation of tubulin chaperone E (TBCE) [161, 162]. Models of many other PNS and CNS disorders were also found to be alleviated, involving other neurotoxins [163–165], mutations [166], metabolic perturbations [51, 158] and non-transecting physical forces such as raised intraocular pressure modelling a major risk factor for glaucoma [167, 168]. However, axons are not or not strongly protected in all disease models by blocking programmed axon death, suggesting these are predominantly driven by other mechanisms. These include SOD1 transgene models of ALS [169, 170], SMA models [171, 172] and some other causes of CMT [173]. Full summaries of which models show protective responses are provided in earlier reviews [153, 174].

The ability to alleviate many disease models by blocking programmed axon death indicates that this pathway contributes to axon loss in these models, not necessarily as an initiating event, nor in isolation from other mechanisms, but at least at some level. However, it is also now clear that aberrant activation of programmed axon death can initiate some axonopathies or increase axon vulnerability to stresses that would not normally kill them. To illustrate this in animal models, *Nmnat2* null mice fail to grow long axons and consequently die at birth with respiratory failure [7, 40]. Mice expressing only 30% as much NMNAT2 as C57BL/6 controls have axons that are more vulnerable to stresses such as vincristine, mitochondrial uncoupling and normal ageing [50, 51, 159]. Importantly, both of these mouse genotypes have human counterparts. Biallelic *NMNAT2* null mutation is associated with a similar, even more severe stillbirth phenotype in humans [175]. Partial *NMNAT2* loss-of-function occurs in an inherited polyneuropathy with neuropathic pain [176], and *NMNAT2* expression level shows wide variation in the human population [177]. Taken together with the observations in mice, this suggests a spectrum of intrinsic axon vulnerability in humans.

Aberrant activation can also occur directly through SARM1. In an exciting, recent development, *SARM1* gain-of-function has been shown to be a statistically significant risk factor in sporadic ALS and to associate with hereditary spastic paraplegia and other motor nerve disorders [178, 179]. Taken together, these studies report twelve different missense or microdeletion variants in 17 patients, altering the inhibitory, N-terminal ARM domain of the protein. All constitutively hyperactivate SARM1 basal NADase activity, and at least five of them to a level 20-fold higher than the wild-type enzyme [179]. Remarkably, this activity in this assay exceeds even that of the NMN-activated wild-type protein many times over, raising important questions about how these individuals survive at all, some with age of onset as late as 70 years [179]. Moreover, their occurrence in sporadic rather than familial cases suggests interaction with other risk factors to produce disease. Nevertheless, neurons expressing these gain-of-function variants are more sensitive to stress in primary culture and die in vivo [178, 179], further indicating their pathogenic role. It will be important now to determine whether SARM1 mediates the apparent contribution of STMN2 depletion to ALS [180, 181] and whether the previously reported GWAS linkage to the *SARM1* locus on chromosome 17 is mediated by SARM1 gene expression level [182, 183].

There is also evidence of toxic hyperactivation of SARM1 in human disease. A downstream metabolite of vacor, a disused rodenticide and nicotinamide analogue, is a potent and direct activator of SARM1 that causes SARM1-dependent axon and neuronal death [50, 51]. Before vacor was banned, individuals who used it in suicide attempts and survived often developed widespread neurological deficits and peripheral nerve axon loss within hours or days [56]. It is extremely likely that SARM1 activation was the major cause of this rapid-onset neuropathy. Although these same individuals also often developed diabetes, the onset of neuropathy within hours suggests it was prior rather than secondary to diabetes, although the later may well have sustained the problem.

Table 1 summarises the accumulating evidence of aberrant activation of programmed axon death in specific human diseases. Together with the more widespread alleviation of animal models of peripheral neuropathies, motor nerve disorders and other conditions [153, 174], this suggests that blocking this pathway will be beneficial in at least a subset of patients with activating genetics or environment in multiple disorders.

**Table 1:** Highlighting the relevance of programmed axon death to human peripheral nerve diseases. For additional summary of animal model data, see Conforti et al. [153]

Disease type	Details	References
Fetal akinesia deformation sequence	Stillbirth with complete absence of skeletal muscle, likely of neurogenic origin, and hydrocephalus, associated with biallelic null mutation of *Nmnat2*	[175]
Polyneuropathy with erythromelalgia	Distal sensory and motor axon loss, painful episodes of erythromelalgia in distal limbs especially following infection, associated with biallelic hypomorphic mutation of *Nmnat2*	[176]
ALS	Late-onset (40–71 y) sporadic ALS, spinal or bulbar onset, often though not always progressing quickly. Associated with monoallelic constitutive hyperactivation of SARM1	[178, 179]
Upper and lower motor nerve disorder	Middle age onset with unilateral leg weakness and wasting, slowly progressing over 25 y with later mild hand weakness and lower limb spasticity. Associated with monoallelic constitutive hyperactivation of SARM1	[179]
Vacor neuropathy	Rapid onset (2 h-3d) lower limb weakness and numbness, ataxia, areflexia, following vacor ingestion. Often associated with additional CNS phenotypes and diabetes. Neurotoxic effect completely dependent on SARM1	[50, 51, 56]

### Repair Schwann Cell Molecular Mechanisms in PNS Diseases

While nerve regeneration in lower vertebrates and small mammals is fairly efficient, the regenerative capacity of human nerves is much poorer. Less than 50% of patients undergoing surgical repairs of injured median or ulnar nerves regain adequate motor or sensory function, in the long term [184]. Furthermore, regenerative functional outcomes deteriorate with increasing age above 40 years; a more proximal lesion site, and thus increasing regenerative distance; and delaying surgical repair for greater than 6 months after trauma [184]. The reason for the deterioration in repair capacity of peripheral nerves appears to be in large part down to the response of Schwann cells [185]. Using nerve grafting experiments in mice, age-related decline in repair capacity of the PNS was found to be due to the age of the nerve graft and not the host, suggesting that the Schwann cell and not the neuronal or inflammatory cell response was responsible [186]. Aged Schwann cells have a reduced capacity to activate myelinophagy and upregulate repair program genes, especially the transcription factor c-JUN [186, 187]. The regenerative decline after delaying nerve repair is also largely due to a loss of regeneration support by repair Schwann cells in the distal stump, rather than a reduction in the intrinsic neuronal regeneration capacity [187–189]. Chronic denervation in mouse and human nerves leads to unfavourable changes in Schwann cells, with downregulation of c-JUN overtime, leading to senescence and eventually cell death [187, 190, 191] (Table 2).

**Table 2:** Highlighting the relevance of the Schwann cell injury response to peripheral nerve diseases

Disease type	Details	References
Chronic denervation after traumatic injury	c-JUN is downregulated in chronic denervation in mouse and human nerves after traumatic injury. This downregulation is correlated with Schwann cell death and regenerative decline Transgenically augmenting c-JUN levels in mouse nerves in chronic denervation rescues regeneration potential	[187, 190, 191]
Inflammatory neuropathies	c-JUN is expression is upregulated in Schwann cells in patients with Guillain–Barre syndrome, chronic inflammatory demyelinating neuropathy and peripheral nerve vasculitis	[192, 193]
Genetic neuropathies	c-JUN is upregulated in Schwann cells in CMT1A patients Deletion of Schwann cell *c-Jun* in a CMT1A mouse model worsens the phenotype, suggesting it is protective for sensory axons NRG1 type 1 is upregulated in postnatal nerves of a CMT1A rodent model Prolonged NRG1 type 1 signalling in a CMT1A mouse model is responsible for onion bulb formation SOX2 and ID2 are upregulated in Schwann cells in a CMT1B mouse model and modulate ER stress	[192, 194–197]
Compression neuropathy	A GWAS of patients with carpal tunnel identified *Adamsts17* as a risk gene. *Adamsts17* is strongly upregulated after nerve injury	[198]

The Schwann cell injury response is also activated in genetic and acquired neuropathies [199] (Table 2), similar to the situation described above for programmed axon death. In particular, c-JUN expression has been identified in Schwann cells in nerves of patients with a form of inherited neuropathy, Charcot-Marie-Tooth disease 1A (CMT1A) and inflammatory neuropathies such as chronic inflammatory demyelinating polyradiculoneuropathy (CIDP), Guillain-Barré syndrome (GBS) and vasculitic neuropathy [192, 193]. Additionally, a recent genome wide association study of carpal tunnel patients revealed that single nucleotide polymorphisms in the *Adamts17* gene, which is upregulated by repair Schwann cells after injury, appears to confer risk to the development of carpal tunnel syndrome, a very commonly occurring compression neuropathy of the median nerve at the wrist [198]. The major question arising from all these findings is whether a partial Schwann cell injury response in the context of neuropathy is a broadly protective or deleterious reaction?

Interestingly, deletion of c-JUN in Schwann cells in a mouse model of CMT1A leads to a more severe phenotype with greater sensory axonal loss [197]. This suggests that c-JUN is upregulated in Schwann cells partially as a protective response in the context of neuropathy. Additionally, both SOX2 and ID2, two inhibitors of myelin differentiation that are also upregulated in repair Schwann cells after injury and in the context of neuropathy, appear to play a protective role. Deletion of either *Sox2* or *Id2*, specifically in Schwann cells, in the mouse model of CMT1B, increases endoplasmic reticulum stress markers and worsens the dysmyelination phenotype [196]. The idea that Schwann cells utilise a partial injury reaction as an initial protective response in neuropathy is further exemplified by the role of NRG-1 type I in CMT1A [194, 199]. NRG-1 type 1 is upregulated by Schwann cells in CMT1A nerves in postnatal development where it helps promote myelination and ameliorate the disease phenotype [194]. Transgenic overexpression of axonally derived NRG-1 or supplementation with soluble NRG-1 in early postnatal development was sufficient to improve the myelination status of axons and compound motor action potentials (CMAPs) on neurophysiological testing in CMT1A rodent models [195]. However, despite the beneficial effects of NRG-1, prolonged Schwann cell NRG-1 type I paracrine signalling in CMT1A actually drives pathological hypermyelination and onion bulb formation. The Schwann cell specific deletion of *Nrg1* in a CMT1A mouse model led to a better clinical phenotype, with improved neurophysiological and neuromuscular function [194]. Thus NRG-1 has a complex role in inherited demyelinating neuropathies, but these studies demonstrate that there may be a therapeutic window for exogenous NRG-1 early on in the disease course for genetic neuropathies such as CMT1A.

The Schwann cell injury response is also relevant to PNS tumours. Dysregulation of crucial pathways that regulate repair Schwann cells appears to be important in the formation of malignant peripheral nerve sheath tumours (MPNST), which are highly aggressive and invasive tumours that originate from the Schwann cell lineage [200]. Interestingly, despite repair Schwann cells adopting an EMT-like gene signature after nerve injury, lineage tracing studies in mice have shown that they remain lineage restricted and are not multipotent [14, 201]. This suggests that molecular regulators of repair Schwann cells likely prevent tumour formation. In these tumours, the HIPPO-TAZ/YAP pathway is hyperactivated, PRC2 is inactivated, and there is a complete loss of the H3K27 trimethylation mark along with inactivation of the Ink4a/Arf locus promoting unrestricted proliferation and malignant transformation [202–206]. Thus, deranged activation of key pathways and molecular regulators in repair Schwann cells plays a central role in tumourigenesis.

## Future Treatment Strategies to Protect Against Axon Loss and Promote Axon Regeneration in the PNS

### Therapeutic Opportunities in Programmed Axon Death

The ability of NMNAT overexpression or SARM1 deletion to delay axon loss and symptoms in animal models of widely varying neurological disorders [153, 174] has long suggested therapeutic potential if drugs could be developed to mimic these effects in patients. While the prospect of enhancing NMNAT activity or expression, or the NMNAT2 stability, requires some novel approaches to drug discovery, the discovery of proteins such as SARM1 and MYCBP2/PHR1 whose activities are required for axons to degenerate presents a seemingly more feasible way to block programmed axon death using inhibitors [9, 10, 12, 207]. Knockdown of SARM1 provides another route to protecting axons [208]. The discovery of SARM1’s NADase activity [12] and the serine-linked ubiquitylation activity of MCYBP2/PHR1 [207], together with relevant structural information for each protein [207, 209], further increase their attractiveness as drug targets. Potential adverse effects of blocking drugs include interference with innate immunity for SARM1 and axon growth effects for MYCBP2/PHR1, but mouse data suggest that with careful targeting and/or timing, these risks could be largely avoided. Other points for intervention in the pathway include MAPK inhibition [210] and supplementation with NAD precursors such as nicotinamide [211], nicotinamide riboside [212] or nicotinic acid riboside.

A very important question is the degree of axon protection that may be feasible with such approaches. For some years, animal model data suggested this may be partial and temporary but applying one single, major stress, such as an overexpressed mutant transgene or a high dose of a toxin, to genetically homogeneous mice does not represent what happens in most human disease. Sporadic disease results from a combination of multiple genetic and environmental risks, and in patients where genetic or toxic activation of programmed axon death is one of these, there is a realistic likelihood that by fully removing that risk pharmacologically, there could be substantial protection. This is particularly demonstrated by the lifelong rescue of the otherwise early lethal phenotype of *Nmnat2* null mice when SARM1 is removed [48] and the full rescue of vacor-treated *Sarm1* null neurons [50, 51]. It follows that it will be important to identify the specific diseases and patients in whom programmed axon death is most activated by genetic or toxic mechanisms as these are the individuals likely to respond best to drugs blocking SARM1 or other pathway components. The recent discovery of such genetic or toxic activation mechanisms [50, 51, 178, 179] are important first steps towards this. Additional opportunities arise in disorders such as chemotherapy-induced peripheral neuropathies (CIPN) where the axonal stress is anyway temporary, so supporting axons through this period could allow full prevention or recovery [213]. Thus, in CIPN, ALS, rare, inherited polyneuropathies or any other disorders in which programmed axon death activation is identified, there are good prospects for long-term beneficial effects.

Another important consideration is whether SARM1 would need to be fully inactivated for a protective effect or only partially inactivated. When its role in axon degeneration was first identified, transected homozygous null axons in mouse sciatic nerve were found to survive for 2–3 weeks, but those of hemizygous nulls were not protected at 5 days [10]. Now, however, it has become clear that hemizygosity, and similar degrees of knockdown achieved with antisense oligonucleotides, is also partially protective against multiple axon stresses including vincristine toxicity and axotomy [213, 214]. Thus, it is reasonable to expect that partial inhibition or silencing of SARM1 in the right patients for the right disease could still be profoundly protective.

### Therapeutic Opportunities in the Schwann Cell Response

There are two substantial problems that need to be addressed in order to successfully promote axon regeneration and functional recovery after human traumatic neuropathy. Firstly, if a nerve is fully transected, a regeneration gap is formed that needs to be bridged either by an autologous nerve graft or by an artificial conduit, if a nerve graft is not suitable. Secondly, repair Schwann cell numbers decline over time in the denervated distal stump, and this adversely affects functional outcomes [190, 191, 215]. One way to overcome these issues is to culture, expand and transplant a patient’s own Schwann cells back into the injured nerve and/or nerve graft/conduit. Two cases of young adults with high sciatic nerve injuries and large regeneration gaps, that normally have poor functional outcomes when treated with sural nerve graft alone, have been treated with combined autologous sural nerve graft and autologous Schwann cell transplant with good regenerative outcomes over a 3-year follow up period [216]. Thus, autologous Schwann cell transplant may afford some promise in improving surgical repair outcomes from traumatic neuropathies.

However, use of autologous Schwann cell transplants does remain constrained by the fact that the Schwann cell supply must come from the patient’s own nerves and that these transplanted Schwann cells, similar to endogenous Schwann cells, will, once transplanted, still lose repair-promoting potential over time and perish. In order to solve the first problem, Schwann cells can be cultured directly from human skin samples [217], or cell reprogramming technology has been used to generate human Schwann cells directly from skin fibroblasts [218–221]. The second problem requires boosting the repair capacity and/or survival of Schwann cells in the injured nerve. One way to achieve this is through neurotrophic factor administration. Application of exogenous GDNF and BDNF protein to various peripheral nerve experimental animal injury models has generally shown positive outcomes. However, this approach, as a treatment strategy in patients, is limited as neurotrophins have short half-lives and penetrate tissues poorly [222]. Gene therapy can be used to abrogate these issues, however, continuous supply of GDNF to a regenerating peripheral nerve through lentiviral delivery results in aberrant sprouting and axon trapping, impairing functional outcomes [223]. Thus delivery of lentiviral expressed GDNF using an immune evasive tetracycline inducible switch, allowing for a pulsed supply of GDNF to the injured nerve, can not only protect motor neurons from injury induced death but also promote long range motor axon regeneration in a spinal root avulsion model [146]. The transcription factor c-JUN is another attractive candidate to improve the PNS repair response in situations where axon regeneration is impaired. These include long-distance regeneration across an artificial conduit to bridge proximal and distal stumps; in chronic denervation; ageing; and to encourage regeneration after secondary axonal loss in inherited and acquired neuropathies [185]. In this regard, Schwann cells transduced with a tetracycline-inducible c-JUN lentiviral construct and then transplanted into a nerve conduit were able to enhance axonal regeneration across a 10 mm gap in rats [224]. Furthermore, overexpression of one allele of c-JUN, specifically in Schwann cells, is able to rescue the decline in axon regeneration rates in aged animals and also after chronic denervation, without appearing to be tumourigenic in mice [89, 187].

It is likely a combination of the above strategies will be required in order to achieve adequate human peripheral nerve regeneration. Furthermore, cell transplant and gene therapy approaches will need to be applied alongside improved engineering of nerve graft conduits, a topic which is reviewed elsewhere [225].

## Future Perspectives: Outstanding Questions

The genetic association of *NMNAT2* and *SARM1* with polyneuropathies and ALS and other motor nerve disorders [175, 176, 178, 179] raises the question of whether coding or gene expression variants in programmed axon death genes influences also other disorders. Certainly, animal model studies suggest so. SARM1 deletion alleviates chemotherapy-induced peripheral neuropathy with vincristine, paclitaxel, bortezomib, cisplatin and oxaliplatin and neuropathy in a type 2 diabetes model using a high fat diet [157, 158, 208, 214, 226, 227], all of which suggest relevance to more common disorders of peripheral nerve. Association with idiopathic peripheral neuropathy, inflammatory neuropathies and other toxic neuropathies, building on findings with vacor as well as cancer chemotherapy drugs will also be important to explore. SARM1 may also have a wider role in ALS than the rare coding variants so far identified, considering the reported GWAS linkage [182] and involvement of programmed axon death regulator STMN2 in this disease [180, 181].

The Schwann cell injury phenotype does appear to vary between motor and sensory nerves and between myelinating and non-myelinating Schwann cells [234, 233]. One future research area will be detailing the transcriptomic and proteomic variation, potentially with single cell resolution, in the repair Schwann cell response between motor and sensory nerves, and between myelinated and unmyelinated fibres, in addition to the effect of different anatomical locations, including root, various different peripheral nerves and end organ associated Schwann cells. This may also start to shed some light upon the reason why the many subtypes of inherited or acquired neuropathy present with involvement of specific and unique patterns of affected nerves, limbs or modalities.

Another part of the Schwann cell injury response that may show promise for development of future therapies for neuropathy is manipulation of Schwann cell myelinophagy. Many acquired and inherited neuropathies are characterised by primary demyelination, and in the Trembler J and C22 mice that model inherited demyelinating neuropathies due to Pmp22 point mutation and overexpression, there is evidence that modulating the level of autophagy in Schwann cells alters pathogenesis [228, 229].

While future cell and gene therapies may show early promise for encouraging axon regeneration after traumatic neuropathy, the next significant challenge will be extending these types of treatments to other acquired (inflammatory and vasculitis) and inherited neuropathies, where there can be substantial secondary axon loss. Any treatment designed to promote axon regeneration will still need to be used in combination with disease modifying treatments, such as the use of immunosuppressive agents in acquired neuropathies and potentially the future use of new genetic therapies for inherited neuropathies.

## Supplementary Information

Below is the link to the electronic supplementary material.

Supplementary file1 (PDF 499 KB)

Supplementary file2 (PDF 499 KB)
